# In vivo evaluation of tumor uptake and bio-distribution of 99mTc-labeled 1-thio-β-D-glucose and 5-thio-D-glucose in mice model

**DOI:** 10.1186/s41181-024-00253-3

**Published:** 2024-03-29

**Authors:** Fabian Muehlberg, Konrad Mohnike, Oliver S. Grosser, Maciej Pech, Juergen Goldschmidt, Karl-Heinz Smalla, Ricarda Seidensticker, Muzaffer Reha Ümütlü, Sinan Deniz, Jens Ricke, Ingo G. Steffen, Osman Öcal, Max Seidensticker

**Affiliations:** 1 Department of Cardiology, MSB Medical School Berlin, Hochschule für Gesundheit und Medizin & HELIOS Hospital Berlin-Buch, Berlin, Germany; 2 Department of Radiology and Nuclear Medicine, University Hospital Magdeburg, Magdeburg, Germany; 3 MVZ DTZ Diagnostisch Therapeutisches Zentrum Am Frankfurter Tor Und MVZ DTZ Diagnostisch Therapeutisches Zentrum Am Onkozentrum Berlin OZB, Berlin, Germany; 4 Research Campus STIMULATE, Otto-Von-Guericke University, Magdeburg, Germany; 5 Leibniz Institute for Neurobiology, Department of Neurochemistry and Molecular Biology, Magdeburg, Germany; 6 Leibniz Institute for Neurobiology, Research Group Neuroplasticity, Magdeburg, Germany; 7 Institute for Pharmacology and Toxicology, Medical Faculty, Otto-Von-Guericke University, Magdeburg, Germany; 8 Center for Behavioral Brain Sciences - CBBS, Otto-Von-Guericke-Universität Magdeburg, Magdeburg, Germany; 9 Department of Radiology, LMU University Hospital, LMU Munich, Munich, Germany; 10 Department of Nuclear Medicine, Charite Universitätsmedizin Berlin, Berlin, Germany; 11 LMU Klinikum München, Klinik Und Poliklinik Für Radiologie, Marchioninistr 15, 81377 Munich, Germany

**Keywords:** Technetium-99 m, Glucose, Carbohydrate, Tumor, SPECT, 1-thio-β-D-glucose, 5-thio-D-glucose, HCT-116, A549

## Abstract

**Background:**

To investigate the capacity of ^99m^Tc-labeled 1-thio-β-D-glucose (1-TG) and 5-thio-D-glucose (5-TG) to act as a marker for glucose consumption in tumor cells in vivo as well as to evaluate the biodistribution of 1-TG and 5-TG. We investigated the biodistribution, including tumor uptake, of 1-TG and 5-TG at various time points after injection (0.5, 2 and 4 h) in human colorectal carcinoma (HCT-116) and human lung adenocarcinoma (A549) xenograft bearing nude mice (N = 4 per tracer and time point).

**Results:**

Ex vivo biodistribution studies revealed a moderate uptake with a maximum tumor-to-muscle ratio of 4.22 ± 2.7 and 2.2 ± 1.3 (HCT-116) and of 3.2 ± 1.1 and 4.1 ± 1.3 (A549) for 1-TG and 5-TG, respectively, with a peak at 4 h for 1-TG and 5-TG. Biodistribution revealed a significantly higher uptake compared to blood in kidneys (12.18 ± 8.77 and 12.69 ± 8.93%ID/g at 30 min) and liver (2.6 ± 2.8%ID/g) for 1-TG and in the lung (7.24 ± 4.1%ID/g), liver (6.38 ± 2.94%ID/g), and kidneys (4.71 ± 1.97 and 4.81 ± 1.91%ID/g) for 5-TG.

**Conclusions:**

1-TG and 5-TG showed an insufficient tumor uptake with a moderate tumor-to-muscle ratio, not reaching the levels of commonly used tracer, for diagnostic use in human colorectal carcinoma and human lung adenocarcinoma xenograft model.

**Supplementary Information:**

The online version contains supplementary material available at 10.1186/s41181-024-00253-3.

## Introduction

Malignant lesions have unique properties related to their origin tissue, but they show a common specification of increased metabolism and cell replication, which leads to increased glucose consumption (Weber 1977; Warburg et al. 1967).

This situation has been exploited for detection of primary and metastatic cancers using positron emission tomography (PET) by injecting tracers mimicking glucose (Rusthoven et al. 2004; Strauss and Conti 1991; Wahl, et al. 1991). The glucose analogue 2-deoxy-2-[^18^F] fluoro-D-glucose (^18^F-FDG) is the most commonly used tracer for PET until today. The increased accumulation of ^18^F-FDG has been shown in a wide range of tumors, and it has been successfully implemented in clinical routine for diagnostic purposes, evaluation of therapy response, and characterization of malignant behavior of lesions (Schelling et al. 2000).

Although it has been a routine procedure, labeling glucose with ^18^F is a complex procedure and necessitates use of costly cyclotrons and advanced PET scanning systems, which limits its use (Berger et al. 2003). Furthermore, decay properties of ^18^F-FDG are suboptimal with half-life (t_½_) of approximately 110 min further decreasing cost-effectiveness (Hubner et al. 1996).

These limitations have led to research efforts to develop radiolabeled carbohydrates that are suitable for single-photon-emission computed tomography (SPECT) imaging mainly using the isotope technetium-99 m (^99m^Tc) which would be an equivalent to PET imaging with ^18^F-FDG (Castelli et al. 2011; Jon et al. 2006; Ozker et al. 1999; Welling and Alberto 2010; Yang et al. 2003). ^99m^Tc-labeled glucose in general is a cost-effective tracer with broad in-house availability and favourable decay properties with a half-life of 6 h.

^99m^Tc-labeled 1-thio-β-D-glucose (1-TG) and 5-thio-D-glucose (5-TG) are promising tracer alternatives in this regard. 1-TG and 5-TG are analogues of natural D-glucose; they share the same stereochemistry but presumably do undergo different biological interactions in vitro and in vivo.

We have investigated in vitro the cellular uptake of 1-TG and 5-TG in a human colorectal carcinoma cell line (HCT-116, ATCC number CCL-185™) and a human lung adenocarcinoma epithelial cell line (A549, ATCC number CCL-247™), both known to be avid for ^18^F-FDG (Seidensticker et al. 2014). We have found a significant uptake and time dependency thereof for both 1-TG and 5-TG. Furthermore, we could show an uptake dependency on glucose and insulin as well as a significant uptake inhibition by cytochalasin B. Although substantial, cellular uptake and individual dependencies were less pronounced for both 1-TG and 5-TG as compared with ^18^F-FDG (Seidensticker et al. 2014). Further on, compartmental cellular analysis revealed a high cellular membrane accumulation of 1-TG and 5-TG with a membrane-to-cytosol ratio of 2.14 and 2.02 for in A549, respectively (Seidensticker et al. 2014). This ratio was 0.08 for ^18^F-FDG in the same analysis suggesting, probably related to steric effects of the molecules, a passage through the GLUT channels is impaired.

The aim of this study was to assess the biological properties of 1-TG and 5-TG in vivo in terms of glucose consumption. In order to do that we performed bio-distribution profiles in HCT-116- and A549-tumor-bearing mice.

## Materials and methods

### Radiolabeling

Radiolabeling has been published previously (Seidensticker et al. 2014). In brief, 1-thio-β-D-glucose and 5-thio-D-glucose were obtained from Sigma-Aldrich® (St.Louis, USA). The glucose derivatives (in mean 5.1 µmol) were labeled with a maximum of 370 MBq [^99m^Tc] pertechnetate. The product was analyzed by high-performance liquid chromatography (HLPC) and thin-layer chromatography (TLC) and formulated in 0.9% NaCl for intravenous administration. The required minimum radiochemical purity for 1-TG and 5-TG was defined as 95%. Detailed analytic methods on radiolabeling process and purity assessment can be found in supplementary data (Additional File 2: Fig. S2 and Additional File 6).

### Tumor model

All experiments were approved by the local animal welfare committee (Landesverwaltungsamt Sachsen-Anhalt; reference number 203.h-42502-2-847 Uni MD), and all methods were carried out in accordance with relevant guidelines and regulations. The reporting in this manuscript follows the recommendations in the ARRIVE guidelines.

HCT-116 colorectal carcinoma cell line and the A549 human lung adenocarcinoma epithelial cell line were obtained from the American type-culture collection (ATCC®, Bethesda, USA). HCT-116 cells were grown in DMEM high-glucose medium, supplemented with 10% fetal bovine serum, 100 IU/ml penicillin and 100 IU/ml streptomycin. A549 cells were cultured using F-12 medium supplemented with 10% fetal bovine serum, 100 IU/ml penicillin and 100 IU/ml streptomycin. Cells were kept in a humidified incubator with 5% CO_2_ at 37° C.

Subculturing was performed using trypsin–EDTA at 70–80% cellular confluence. Tissue culture material, medium, serum and supplements were obtained from CellGro® (Manassas, USA). Only cell passages 4 to 6 were used for subsequent in vivo experiments.

For in vivo studies, 6- to 8-weeks-old athymic nu/nu female mice were obtained from Charles River Laboratories (L’Arbresle, France).

A total of 48 mice has been used in the biodistribution analysis of this study, being 4 animals for each two cell lines, two tracers, and three time points of evaluation. Mice were kept in individually ventilated cages under standard conditions with food and water ad libitum. At the time of tumor cell inoculation, cells of the corresponding tumor cell line (HCT-116 or A549) were suspended at a concentration of 40 × 10^6^ cells/ml. In total, 2–3 × 10^6^ cells were injected subcutaneously into the back of each mouse. Biodistribution and imaging analysis were performed after 3–4 weeks, when the tumor was palpable.

### *In vivo* biodistribution studies

After fasting for 4 h mice were injected via a lateral tail vein with 1-TG (30 ± 11.7 MBq) or 5-TG (26 ± 11.7 MBq) under isoflurane anesthesia (1%–2% isoflurane in 2:1 O_2_:N_2_O volume ratio) and sacrificed 30 min, 2 h and 4 h later. Mice were under persistent anesthesia and heating throughout the entire period of accumulation. For each time point, tracer and 4 tumor bearing mice animals were examined. Blood, urine, organs, and tumors were collected and weighed. For tumor specimen, liquid necrotic areas were discarded and only solid tumor parts were examined. Activity content was assessed by well counter measurement. Tissue counts and injected dose for individual mice were decay-corrected to the time of euthanasia. Tissue uptake was expressed as the percentage injected dose per gram of tissue (%ID/g). Values of tumor uptake has been divided by muscle uptake for each animal to calculate tumor-to-muscle ratio.

### SPECT/CT imaging

A total 4 mice were scanned as exemplary cases for in vivo SPECT/CT imaging. Under isoflurane anesthesia (1%–2% isoflurane in 2:1 O2:N_2_O volume ratio) tracer injection and whole-body imaging was performed 2 h and 4 h after injection with a 4-head NanoSPECT/CT scanner (Mediso Ltd.). SPECT scans were acquired using a multipinhole collimator set (SCIVIS), each collimator with 9 pinholes (1.4-mm pinhole diameters). SPECT imaging was performed with an energy window of 140 keV ± 5% and an acquisition time of 60 min. Images were reconstructed with isotropic voxel output sizes of 300–448 µm, depending on count rates (HiSPECT; SCIVIS). CT scans were acquired at 98 µm detector resolution and reconstructed at isotropic voxel sizes of 200 µm (InVivoScope 1.43; Bioscan). Further on, six non-tumor bearing mice were scanned to qualitatively evaluate the thyroid uptake of tracers.

### Data analysis and statistics

All statistical analyses were performed using R statistical and computing software, version 3.5.0 (http://www.r-project.org, R Foundation for Statistical Computing). Results were presented as the mean (standard deviation). Independent samples of tumor uptake for each organ, as well as for tumor tissue and tumor-to-muscle ratio, tracer uptake at each timepoint were compared with Kruskal Wallis H test, and Mann–Whitney-U test was used as post-hoc test in pairwise comparisons of variables with significant results. Two-sided *p*-values of < 0.05 were considered statistically significant.

## Results

### Biodistribution and tumor uptake

**Fig. 1:**
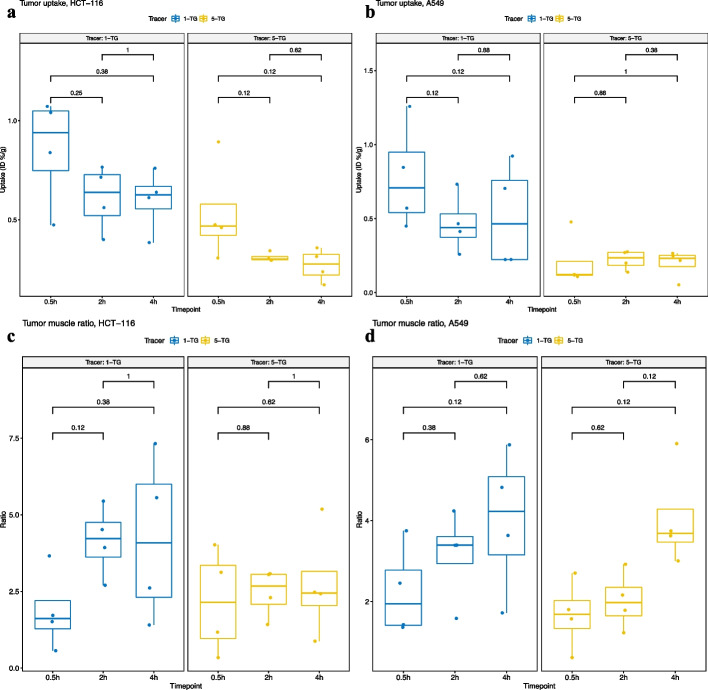
**a**–**d** Tumor uptake and tumor-to-muscle ratio of 1-TG and 5-TG in HCT-116 and A549 over time (by tracer). *p*-values are given for comparison of each time point

*In-vivo* biodistribution data shows moderate specific tumor uptake of both tracers in both tumor cell lines (Table 1, Additional File 5: Table S1 on individual cell lines and Fig. 1a–d and Additional File 1: Fig. S1 a–d online).

**Table 1:** Organ and tissue uptake of 1-TG (a) and 5-TG (b) over time

(a) 1-TG							
	0.5 h		2 h		4 h		
Organ	ID%	SD	ID%	SD	ID%	SD	p-value
Blood	2.34	1.14	0.99	0.35	0.51	0.16	**0.0006**
Brain	0.07	0.04	0.04	0.02	0.02	0.01	**0.002**
Heart	0.87	0.27	0.52	0.18	0.31	0.11	**0.0006**
Liver	2.60	2.80	2.34	2.67	1.20	0.73	0.547
Kidney left	10.10	3.03	8.28	4.49	12.18	8.77	0.564
Kidney right	9.82	3.13	8.43	4.58	12.69	8.93	0.547
Spleen	0.93	0.71	0.85	0.86	0.56	0.47	0.329
Stomach	0.29	0.07	0.18	0.10	0.20	0.13	0.080
Bowel	0.67	0.27	0.68	0.44	0.33	0.28	**0.040**
Lungs	1.44	0.53	0.82	0.25	0.54	0.14	**0.001**
Muscle	0.46	0.19	0.15	0.03	0.16	0.11	**0.001**
Tumor	0.82	0.30	0.54	0.18	0.56	0.26	0.1
Tumor-to-muscle-Ratio^#^	2.06	1.14	3.65	1.18	4.12	2.12	**0.001**
Tumor (A549)*	0.78	0.36	0.47	0.20	0.52	0.35	0.397
Tumor-to-muscle-Ratio (A549)*	2.25	1.12	3.15	1.12	4.01	1.78	0.210
Tumor (HCT-116)*	0.86	0.27	0.61	0.16	0.60	0.16	0.231
Tumor-to-muscle-Ratio (HCT-116)*	1.87	1.30	4.15	1.15	4.23	2.70	0.167

(b) 5-TG							
	0.5 h		2 h		4 h		
Organ	ID%	SD	ID%	SD	ID%	SD	p-value
Blood	1.43	1.12	0.70	0.28	0.51	0.23	*0.060*
Brain	0.11	0.11	0.07	0.05	0.05	0.03	0.505
Heart	0.85	0.43	0.42	0.10	0.29	0.14	**0.024**
Liver	6.38	2.94	7.37	4.83	5.95	2.63	0.898
Kidney left	4.71	1.97	3.34	1.31	2.25	0.91	*0.059*
Kidney right	4.81	1.91	3.40	1.33	2.43	0.90	**0.046**
Spleen	2.90	2.36	3.81	3.17	3.51	2.02	0.796
Stomach	0.46	0.47	0.16	0.10	0.32	0.37	0.394
Bowel	0.64	0.43	0.48	0.48	0.27	0.18	0.172
Lungs	7.24	4.10	3.40	3.01	2.08	1.49	**0.032**
Muscle	0.46	0.87	0.13	0.05	0.11	0.13	0.119
Tumor	0.37	0.27	0.27	0.07	0.23	0.09	0.505
Tumor-to-muscle-Ratio^#^	1.92	1.27	2.25	0.73	3.41	1.60	0.134
Tumor (A549)*	0.21	0.18	0.22	0.07	0.20	0.10	0.550
Tumor-to-muscle-Ratio (A549)*	1.67	0.86	2.03	0.71	4.07	1.26	**0.023**
Tumor (HCT-116)*	0.53	0.25	0.31	0.02	0.27	0.08	*0.086*
Tumor-to-muscle-Ratio (HCT-116)*	2.16	1.70	2.46	0.78	2.74	1.79	0.981

Bold indicates p < 0.05

n = 8 (A549 plus HCT116) if not indicated otherwise

*n = 4, and ratio instead of ID%/g

#Both cell lines, and ratio instead of ID%. p-values are given for comparison of each time point for each tissue. Only mice with tumor inoculation have been used in this analysis

Tumor uptake of 1-TG peaked at 30 min with 0.86 ± 0.27%ID/g (HCT-116) and 0.78 ± 0.36%ID/g (A549). However, 1-TG displayed the highest tumor-to-muscle ratio at 4 h (4.2 ± 2.7 for HCT-116 and 4.0 ± 1.8 for A549) versus the tumor-to-muscle ratio at tumor uptake peak at 30 min of 1.87 ± 1.3 for HCT-116 and 2.3 ± 1.1 for A549; indicating fast initial uptake followed by slow tumor activity washout paralleled by a stronger washout in the muscle (Table 1a).

Tumor uptake dynamics of 5-TG were similar to 1-TG with a peak tumor uptake at 30 min with 0.53 ± 0.25%ID/g (HCT-116) and at 2 h with 0.22 ± 0.07%ID/g (A549). Tumor-to-muscle ratio peaked at 4 h (4.1 ± 1.3 with A549 and 2.7 ± 1.8 with HCT-116) versus the tumor-to-muscle ratio at tumor uptake peak at 30 min of 1.7 ± 0.9 with A549 and 2.2 ± 1.3 with HCT-116; indicating a similar kinetic of tumor and muscle as compared to 1-TG (Table 1b).

Still, tumor-to-muscle-ratios were greater than 1 at all time points for both tracers (see Table 1).

### Comparison between the cell lines

Although HCT-116 had higher uptake for both tracers at each time point, there was a significant difference only for 5-TG at 2 h (*p* = 0.029). The difference between the cell lines diminished through expression by tumor-to-muscle ratio, for both tracers (Additional File 1: Fig. S1 a-d online).

### Comparison of the tracers

1-TG had more intense uptake for both cell lines at all time points compared to 5-TG, but the difference was only significant for HCT-116 at 2 and 4 h, and in tendency significant for A549 at 0.5 h (*p* = 0.057). Although this trend was also preserved in tumor-to-muscle ratio, the difference was not significant at any time point (Table 1 and Fig. 1). Furthermore, there was a significantly stronger muscle background in 1-TG as compared to 5-TG (p = 0.01), not shown in tables.

### Tracer uptake tumor dynamics

Tumor uptake of 1-TG was highest at 0.5 h in both cell lines.

Except for the slight increase in uptake of 5-TG in A549 from 0.5 to 2 h, the highest uptake was in the early phase with a decrease at 2 and 4 h for both tracers.

While tumor-to-muscle ratio for 5-TG was stable over time in HCT-116, there was a significant increase at 4 h in A549. Of note, muscle intensity dropped over time in 5-TG as well, but not significantly. Although the difference was not statistically significant between any time points, the tumor-to-muscle ratio for 1-TG had an increasing trend with time (Table 1 and Fig. 1).

### Normal tissue biodistribution

**Fig. 2:**
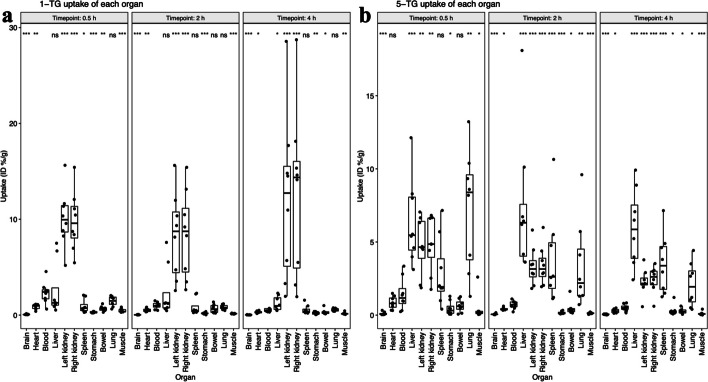
Organ and tissue uptake of 1-TG (**a**) and 5-TG (**b**) compared to blood over time. p-values are given for comparison of each tissue with blood

Details for normal tissue distribution of tracers in non-tumorous tissues of the mice with tumor inoculation can be found in Table 1, Additional File 5: Table S1, and Fig. 2.

1-TG displayed extensive kidney uptake at all time points peaking at 4 h with 12.18 ± 8.77%ID/g and 12.69 ± 8.93%ID/g (left and right kidney, respectively), suggesting a major renal clearance of 1-TG. Besides kidney, only liver showed a significantly higher uptake than blood at 4 h (Fig. 2a).

Kidney uptake was highest at 30 min for 5-TG (4.71 ± 1.97%ID/g and 4.81 ± 1.91%ID/g for left and right kidney, respectively) and decreased towards 4 h (2.25 ± 0.91%ID/g and 2.43 ± 0.9%ID/g). Liver uptake of 5-TG was significantly higher than blood for all time points, peaking at 2 h. Similar uptake dynamics of 5-TG were also noted for the spleen. Notably, pulmonary uptake of 5-TG was higher than blood at all time points with a decreasing trend over time (Fig. 2b).

Biodistribution patterns for blood, heart, stomach, bowel, and muscle were similar for both 1-TG and 5-TG (Table 1 and Additional File 5: Table S1).

### SPECT/CT Imaging

**Fig. 3:**
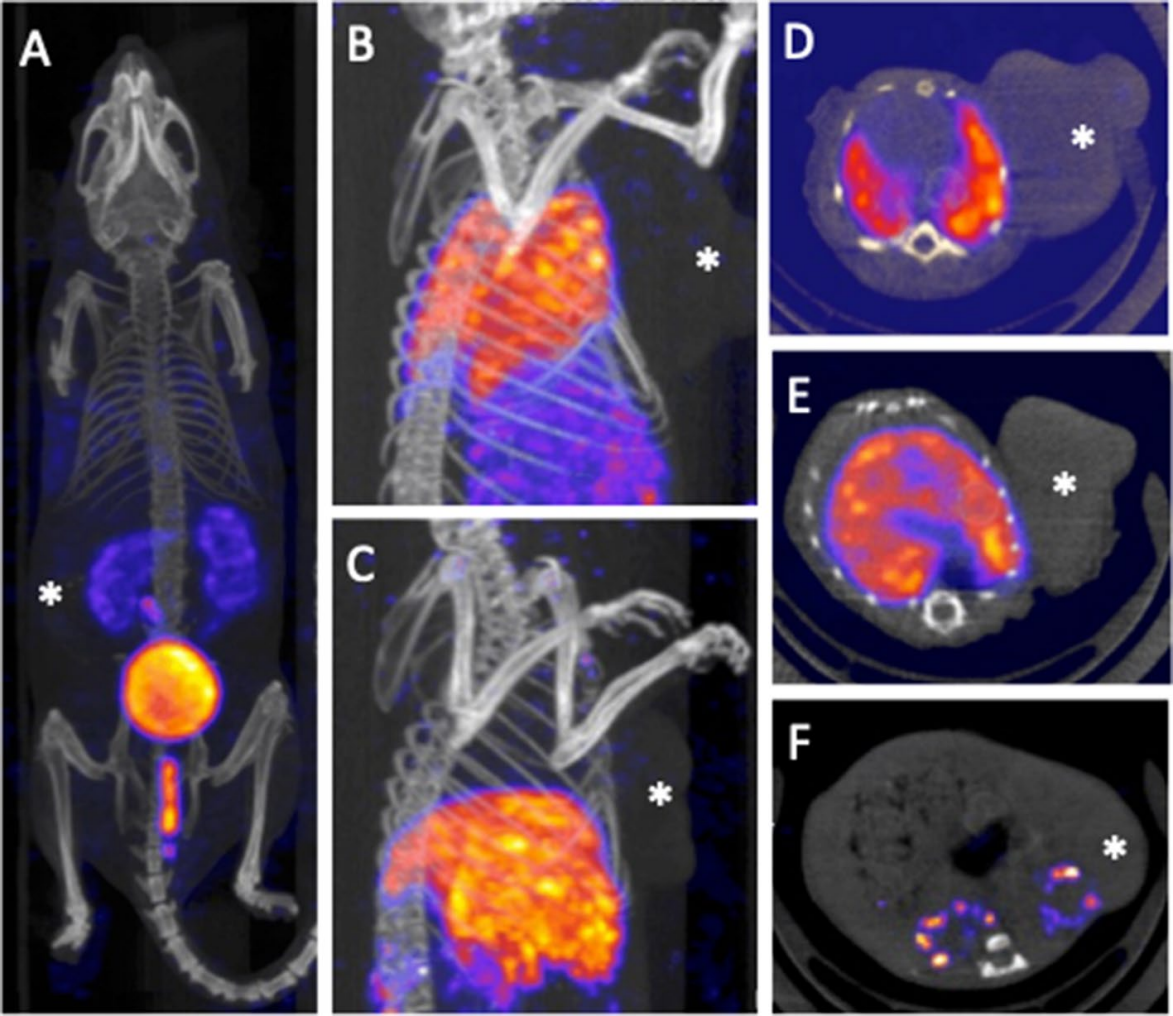
**A** Showing whole body scan 2 h after injection of 1-TG (A549 tumor bearing mouse) with good visibility of renal accumulation; no visible uptake of the tumor in the right back. **B** and **C** showing partial body scan 0.5 h (**B**) and 4 h (**C**) after injection of 5-TG (HCT-116 tumor bearing mouse) with visualization of the lung accumulation (**B**) in the early phase and an increasing liver accumulation over time (**B** and **C**); no visible uptake of the tumor in the left flank. **D** to **F** show axial slices of 5-TG at 0.5 h (HCT-116), 5-TG at 4 h (HCT-116) and 1-TG at 4 h (HCT-116), respectively. Tumor marked with a white asterixis

Imaging confirmed the high renal accumulation in 1-TG as well as the high liver and lung accumulation in 5-TG. For example. a whole-body scan of the exemplary case of A549 tumor-bearing mouse showed good visibility of renal accumulation 2 h after injection of 1-TG but no perceptible uptake of the tumor. Images of another case of HCT-116 tumor-bearing animal 0.5 h and 4 h after injection of 5-TG showed uptake in both lungs and increasing liver accumulation over time without apparent uptake of the tumor (Fig. 3).

## Discussion

Despite tremendous improvements over the past decades, there is still an immanent need for more cost-effective and accessible alternatives to ^18^F-FDG in non-invasive tumor diagnostics and follow-up, especially in developing and emerging countries. In a recent in vitro study, we showed that the ^99m^Tc-labeled glucose derivatives 1-TG and 5-TG display a high uptake in the tumor cell lines HCT-116 and A549 (Seidensticker et al. 2014).

Hence, the present study was designed to investigate bio-distribution and tumor uptake patterns of 1-TG and 5-TG with the same tumor cell lines in vivo.

We were able to show a significant, but moderate tumor uptake in both cell lines and a time dependency thereof for both tracers with more pronounced uptake of 1-TG, a different clearance mechanism for 1-TG (predominantly renal) as compared to 5-TG (predominantly hepatic) and a significant pulmonary and splenic accumulation for 5-TG. A possible reason behind the different clearance mechanisms and biodistribution of these tracers could lie in their molecular structure. Unlike 5-TG, 1-TG contains glucose rings similar to 18F-FDG (Seidensticker et al. 2014).

Albeit we showed a substantial tumor uptake in vivo with tumor-to-muscle ratio of up to four times for 1-TG (at 4 h after injection), the uptake was less pronounced for 1-TG and 5-TG compared with ^18^F-FDG for which previous studies using similar cell lines revealed tumor-to-muscle-ratios of 6:1 to 13:1 (Wang et al. 2016; Lee et al. 2009).

This is in line with our in vitro work and could be a result of lower affinity of 1-TG and 5-TG to GLUT channels as compared to ^18^F-FDG. The higher affinity of ^18^F-FDG is potentially caused by the substitution of hydroxide groups of the glucose ring in the tracer molecule, which increase carrier affinity (Barnett et al. 1973). Further on, steric effects of ^99m^Tc are assumed to probably impair binding to GLUTs. Our in vitro work has also shown that decrease in tracer accumulations of 1-TG and 5-TG in high glucose and Cytochalasin B concentrations were considerably lower than decrease in 18F-FDG accumulation. This also indicates that uptake of 1-TG and 5-TG are potentially regulated mostly by non-glucose dependent mechanisms.

Regarding the different tracer accumulation in the tumors of the two investigated cell lines it can be speculated that it might be attributed to a different proliferation rate, a different vascularization, or a different GLUT-1 expression.

There is limited published in vitro and in vivo data for 1-TG and 5-TG. Our previous in vitro work and the work of Jun Oh et al. revealed substantial in vitro uptake of 1-TG for several carcinoma cell lines (A549, HCT-116, Hs683 and SNU-C5), however, there has been no in vivo study so far to compare our results with.

In contrast to that, for 5-TG there is no in vitro data, but Ozker et al. evaluated tumor uptake in vivo, showing a tumor-to-muscle ratio of up to 4:1 for mice bearing the colon cancer cell line MC26 (Ozker et al. 1999). In the present study this ratio could only be met for A549 cell line at four hours after tracer injection, but not for the colon cancer cell line HCT-116 (maximum tumor-to-muscle ratio of 2.74 ± 1.79, see Table 1), indicating variability of uptake ratios for different cancer types for 5-TG.

Using in vivo biodistribution studies, we showed an extensive renal uptake of 1-TG which peaked at four hours after administration at more than 12%ID/g tissue. This suggests a renal excretion mechanism for this tracer similar to ^18^F-FDG (Akers et al. 2016).

Interestingly, 5-TG showed substantial renal uptake at 30 min (rapidly decreasing at later time points), however, displayed additional extensive hepatic uptake, which peaked at 2 h after injection indicating a more pronounced hepatic accumulation or elimination of the tracer as compared to 1-TG. A more hepatic elimination or accumulation of 5-TG could be the result of several factors: (a) a more pronounced metabolization and degradation by macrophages in the mononuclear phagocyte system (as indicated additionally by high splenic uptake) or (b) due to varying affinities to different GLUT transporters due to molecular structural differences (see above). GLUT2 is markedly expressed on hepatocytes and mediates the facilitated transport of glucose across the cell membranes (Karim et al. 2012). This may lead to hepatic accumulation of 5-TG, if its affinity to GLUT2 is increased as compared to 1-TG. There is only limited data on 5-TG and GLUT2. However, Wang et al. demonstrated that 5-TG prevents streptozotocin induced reduction of GLUT2 transporters in mice indicating its affinity to the transporter (Wang and Gleichmann 1995). Albeit partial biliary excretion of 5-TG is possible, we did not find an accumulation in gut specimen, weakening this hypothesis or indicating an entrapment within the hepatocyte without further biliary excretion. Nevertheless, the more pronounced hepatic accumulation or elimination of 5-TG is yet to be understood. Furthermore, we demonstrated relatively high pulmonary and splenic tracer accumulation of 5-TG. Both organs typically display relatively high glucose consumption in a physiologic setting and are, hence, thought to bind considerable amounts of GLUT-mediated tracers. This is also known for ^18^F-FDG, whose splenic uptake is considered normal if it is less pronounced than the average hepatic uptake which was also shown by our results (Liu 2009). However, lack of strong uptake of 1-TG and 5-TG in brain and heart, which show strong ^18^F-FDG uptake, indicates 1-TG and 5-TG are poor biological mimics of glucose. We speculate that the membrane-dominant accumulation we described previously *in-vitro* could be the cause of a missing passage through the blood–brain barrier (Seidensticker et al. 2014).

As stated in material and methods, a radiochemical purity of > 95%, as confirmed by HLPC and/or TLC, was mandatory for utilization of the labeled tracers in in-vivo experiments (see Additional File 6). Further on, in a previous analysis of our working group, we confirmed the stability of ^99m^Tc labeled 1-TG and 5-TG after 24 h incubation in PBS or bovine serum. The stability and radiochemical purity were high at 99.6–100% in either PBS or serum (Seidensticker et al. 2014) (Additional file 3). Thus, although it is not possible completely eliminate the in vivo instability of the tracers, in vitro analysis supports ruling out that unstable compounds lead to unspecific tracer accumulations that bias results. We have not measured the accumulation of tracer/possibly free ^99m^Tc in the thyroid gland. However, in an experiment of Ozker et al. on the biodistribution of 5-TG, using the same radiolabeling method as in our analysis, no uptake in the thyroid gland was detected (Ozker et al. 1999). Biodistribution measurements with planar imaging in non-tumor-bearing mice support these findings (Additional File 4: Fig. S4 online).

Looking at the biodistribution pattern from the clinical perspective, an only moderate tumor cell uptake and, hence, comparably low visibility in SPECT imaging may limit the diagnostic performance of 1-TG and 5-TG. Furthermore, the tumor-to-blood ratio of 1-TG and 5-TG was relatively low around 1 as compared to ^18^F-FDG which is typically two to three-fold (Hoff et al. 2013). Additionally, SPECT imaging comes along with an intrinsic lower spatial resolution as compared to PET imaging which inflicts imaging with low specific uptake even further. SPECT/CT imaging examples support this with very low visual signals within the tumors (Fig. 3). Interestingly, there is a report on imaging after application of 1-TG in lymphoma patients (using SPECT/CT). A detectable tracer uptake was described. A possible cause for the depicted potential tumor uptake could be a higher intrinsic glucose consumption and a rather solid tumor growth of the evaluated tumors as compared to the in part central necrotic tumors that were evaluated in this analysis (Chernov et al. 2022).

As a limitation, only two commercially available tumor cell lines were investigated in this study, limiting the generalizability of the results. It is possible that different tumor entities would reveal more pronounced tracer uptake (Chernov et al. 2022). However, metabolic active and ^18^F-FDG avid tumor cell lines were chosen to prevent false negative interpretations of the performance of 1-TG and 5-TG to display glucose consumption. However, the chosen cell lines are highly proliferative and show tumor necrosis at an early stage in tumor growth. This might result in an underestimation of tracer uptake. However, central necrotic areas of the tumor specimen were discarded before analysis. Thus, we do not believe that presence of tumor necrosis biased our tracer uptake assessments. On the other hand, presence of central necrosis might influence imaging and might be the cause of contradictory imaging findings to reports of visible 1-TG uptake in rather solid tumors like lymphoma (Chernov et al. 2022). Lack of comparison with ^18^F-FDG-PET as a reference poses an additional limitation. However, our group quantified ^18^F-FDG *in-vitro* consumption of HCT-116 and A549 as compared to 1-TG and 5-TG previously, and additional *in-vivo* experiments do not enhance the main findings of this project. Finally, the lack of quantitative assessment of SPECT/CT images might be regarded as a limitation. However, the focus was set on highly specific and exact measurements of tissue accumulation and we believe imaging on exemplary cases is sufficient, especially in light of the low accumulation of the tracer in tumor tissue. In this scenario, additional imaging experiments do not seem to be justified.

## Conclusions

The ^99m^Tc-labeled glucose derivatives 1-TG and 5-TG showed only moderate uptake in the tumor cells HCT-116 and A549 in vivo. Excretion appears to be more renal-dominant in 1-TG and more hepatic-dominant in 5-TG.

Tumor-to-muscle ratios could not reach levels typical for ^18^F-FDG using HCT-116 and A549. Given these results, 1-TG and 5-TG do not provide sufficient tumor uptake for diagnostic use. However, further research in cost-effective tracer development should be encouraged.

### Supplementary Information

**Additional file 1**. Uptake and Tumor-to-Muscle Ratio by tracer, time and tumor type.

**Additional file 2**. Thin-layer chromatography of ^99m^Tc-labeled 1-thio-β-D-glucose (A) and ^99m^Tc-labeled 5-thio-D-glucose (B). The radiochemical purity is 97.23% (A) and 99.63% (B).

**Additional file 3**. Thin-layer chromatography of ^99m^Tc-labeled 1-thio-β-D-glucose (A+B) and ^99m^Tc-labeled 5-thio-D-glucose (C+D) after 24h incubation at 37°C in phosphate-buffered saline (PBS) (A+C) or bovine serum (B+D), activity 10MBq. The radiochem was >99% in all probes.

**Additional file 4**. Planar imaging 60min p.i. of ^99m^Tc-labeled 1-thio-β-D-glucose (A-C) and ^99m^Tc-labeled 5-thio-D-glucose (D-F) in C57/BL6 non-tumor-bearing mice. No uptake in the thyroid gland visible.

**Additional file 5**. Organ and tissue uptake by tracer and tumor type.

**Additional file 6**. Radiolabeling process.

## Data Availability

The datasets used and/or analysed during the current study available from the corresponding author on reasonable request.
