# Supplementary material for: In vivo evaluation of tumor uptake and bio-distribution of 99mTc-labeled 1-thio-β-D-glucose and 5-thio-D-glucose in mice model
Source: EJNMMI Radiopharm Chem. 2024 Mar 29;9:26. doi: 10.1186/s41181-024-00253-3 (PMC10980667; doi:10.1186/s41181-024-00253-3)

Supp. figure 1a. 1–TG tumor uptake by tumor type

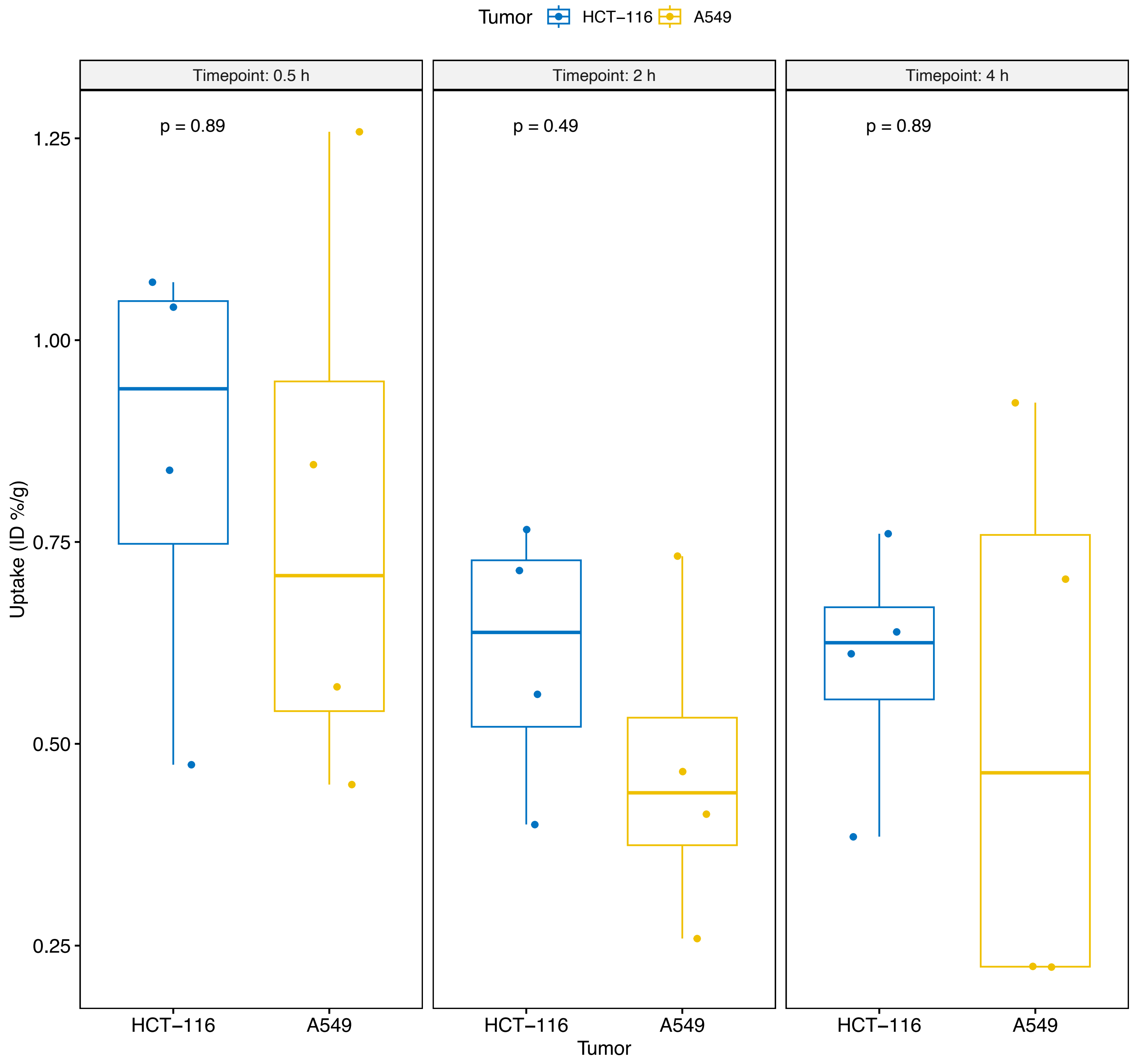

Supp. figure 1b. 5–TG tumor uptake by tumor type

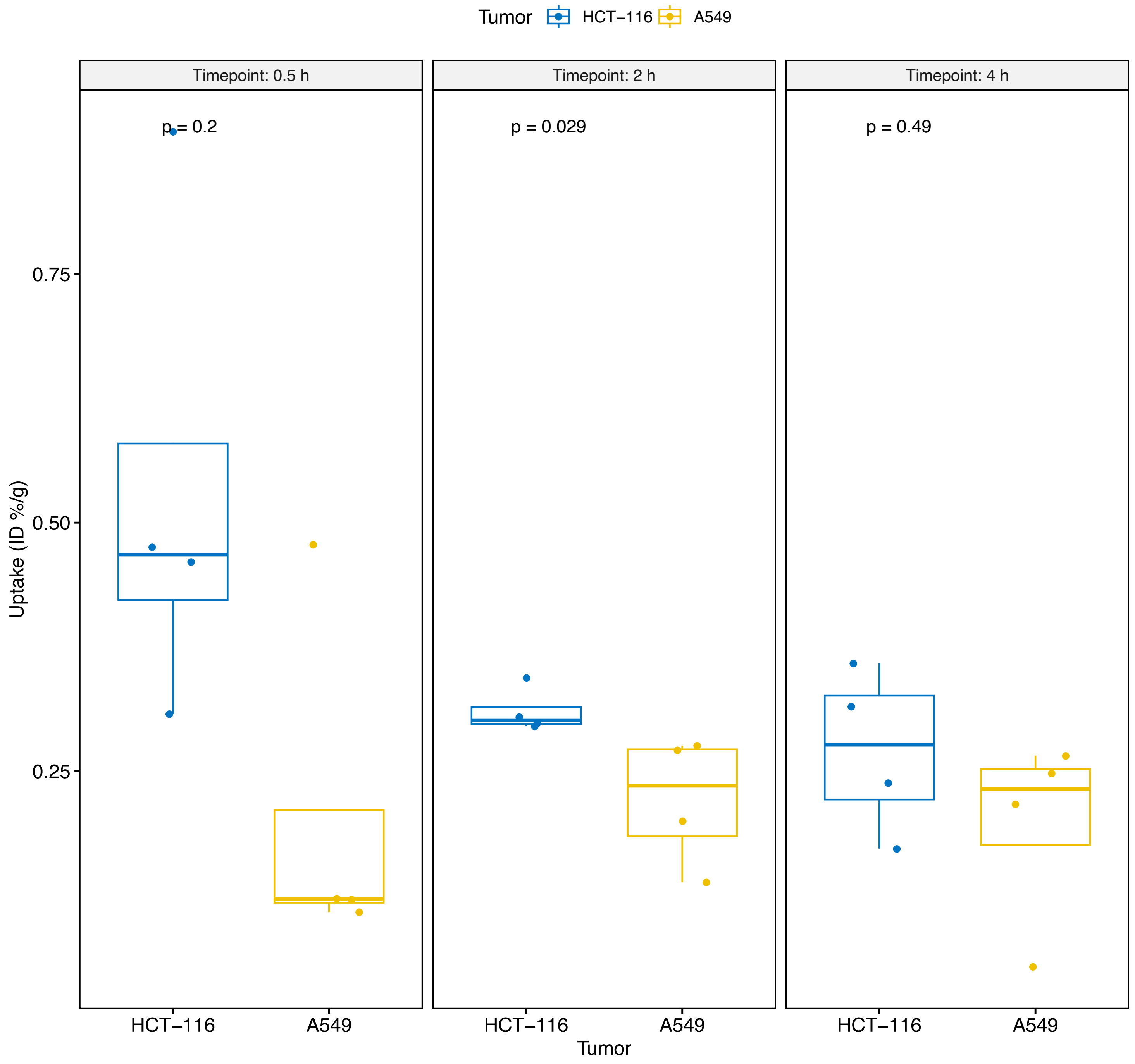

Supp. figure 1c. 1–TG tumor/muscle ratio by tumor type

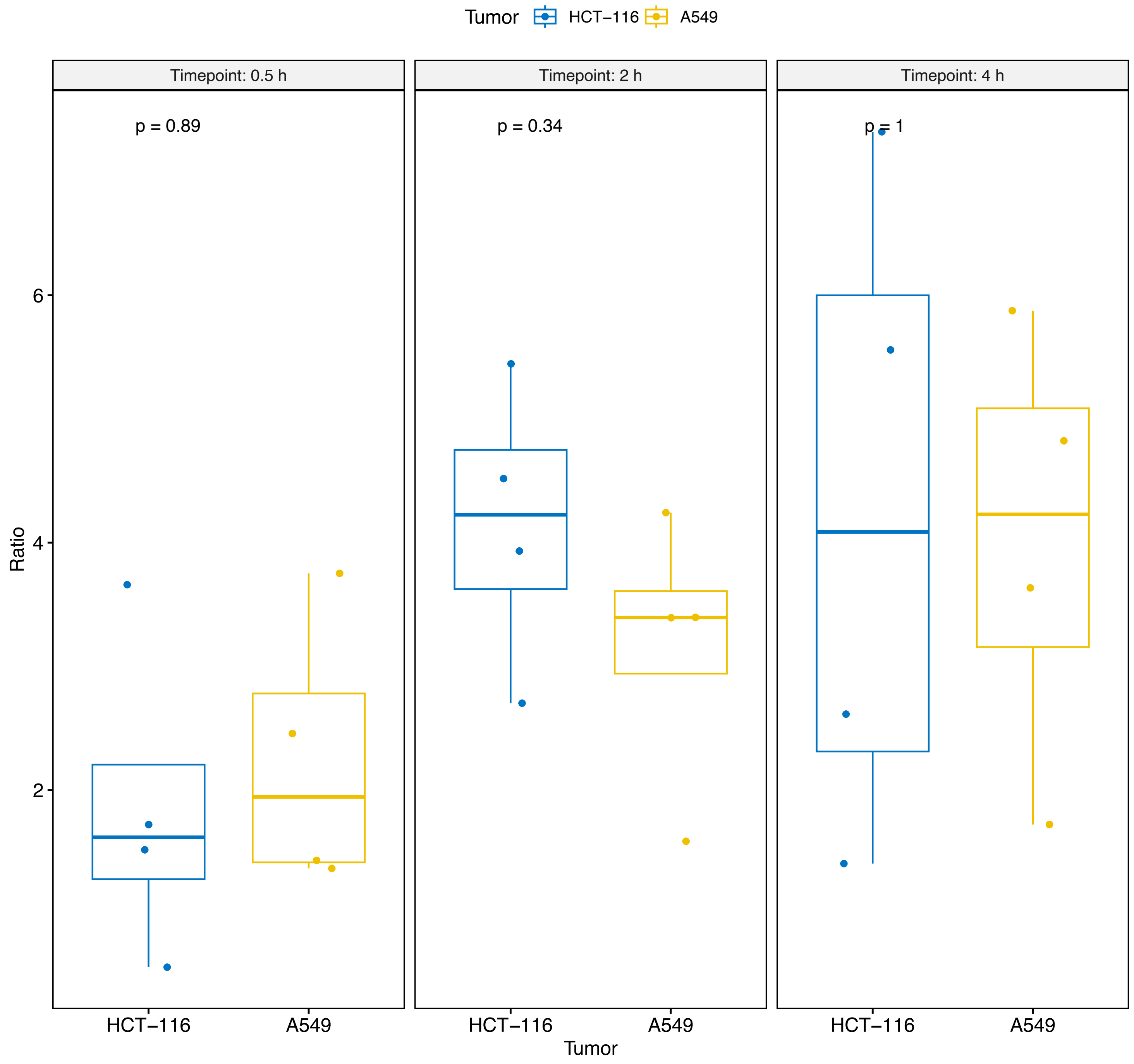

Supp. figure 1d. 5–TG tumor/muscle ratio by tumor type

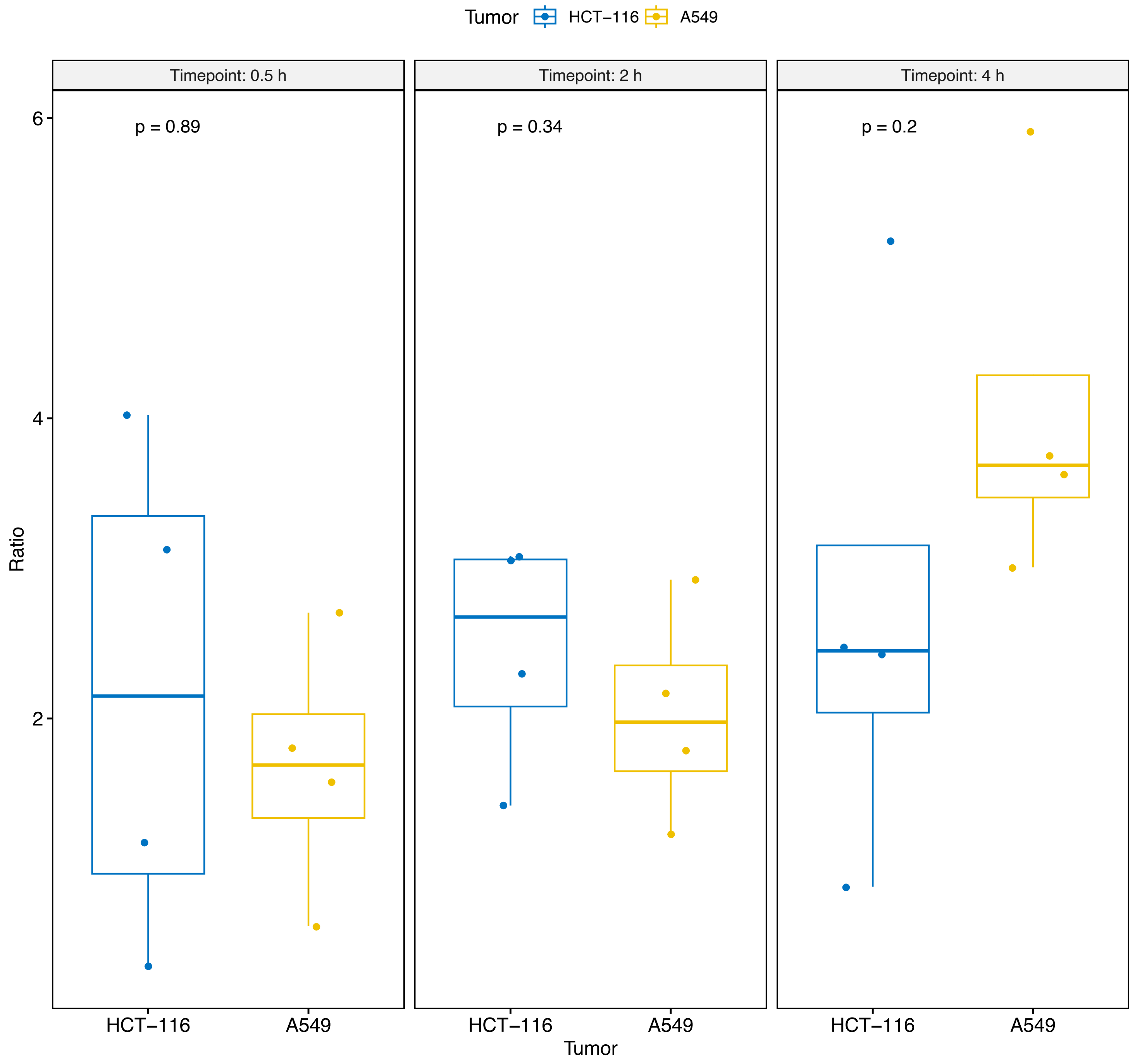

Supplement: Supplementary file 1 — Additional file 1. Uptake and Tumor-to-Muscle Ratio by tracer, time and tumor type. [file 41181_2024_253_MOESM1_ESM.pdf]
