# Supplementary material for: In vivo evaluation of tumor uptake and bio-distribution of 99mTc-labeled 1-thio-β-D-glucose and 5-thio-D-glucose in mice model
Source: EJNMMI Radiopharm Chem. 2024 Mar 29;9:26. doi: 10.1186/s41181-024-00253-3 (PMC10980667; doi:10.1186/s41181-024-00253-3)
Supplement: Supplementary file 2 — Additional file 2. Thin-layer chromatography of 99mTc-labeled 1-thio-β-D-glucose (A) and 99mTc-labeled 5-thio-D-glucose (B). The radiochemical purity is 97.23% (A) and 99.63% (B). [file 41181_2024_253_MOESM2_ESM.pdf]

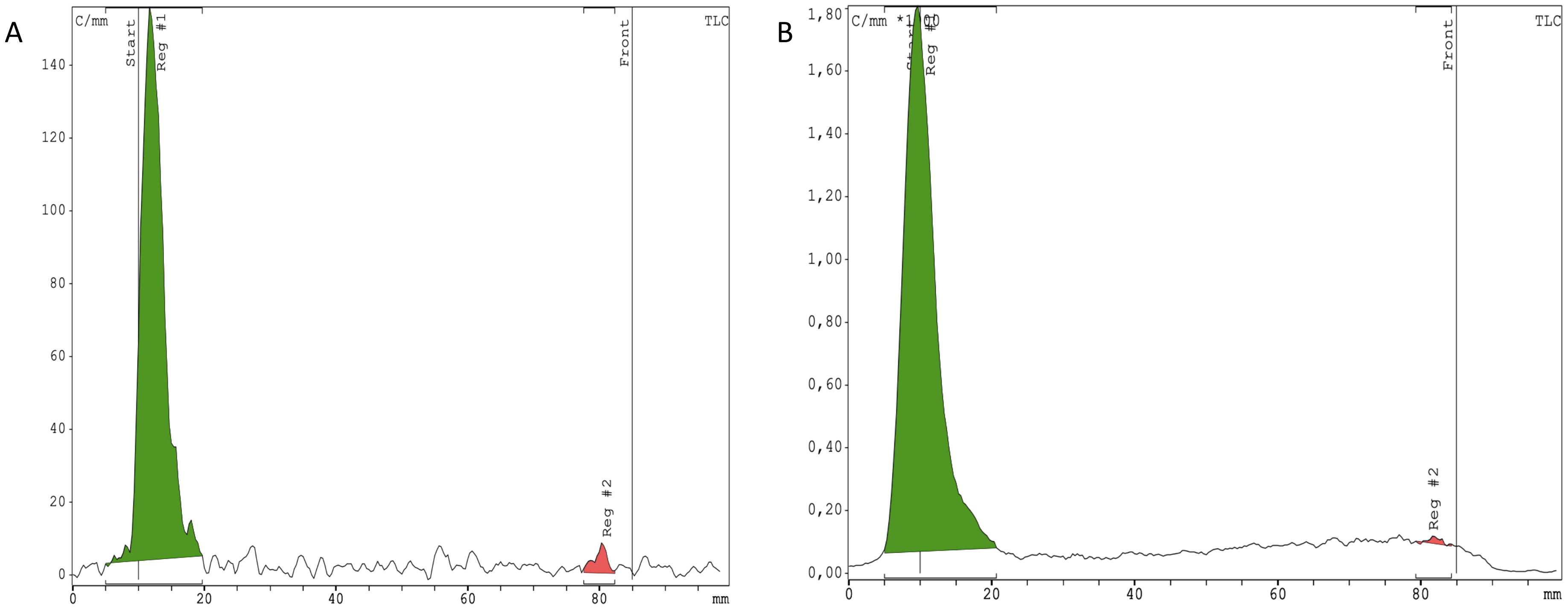

Thin-layer chromatography of ^{99m}Tc-labeled 1-thio-β-D-glucose (A) and ^{99m}Tc-labeled 5-thio-D-glucose (B). The radiochemical purity is 97.23% (A) and 99.63% (B).
