# Supplementary material for: In vivo evaluation of tumor uptake and bio-distribution of 99mTc-labeled 1-thio-β-D-glucose and 5-thio-D-glucose in mice model
Source: EJNMMI Radiopharm Chem. 2024 Mar 29;9:26. doi: 10.1186/s41181-024-00253-3 (PMC10980667; doi:10.1186/s41181-024-00253-3)
Supplement: Supplementary file 3 — Additional file 3. Thin-layer chromatography of 99mTc-labeled 1-thio-β-D-glucose (A+B) and 99mTc-labeled 5-thio-D-glucose (C+D) after 24h incubation at 37°C in phosphate-buffered saline (PBS) (A+C) or bovine serum (B+D), activity 10MBq. The radiochem was >99% in all probes. [file 41181_2024_253_MOESM3_ESM.pdf]

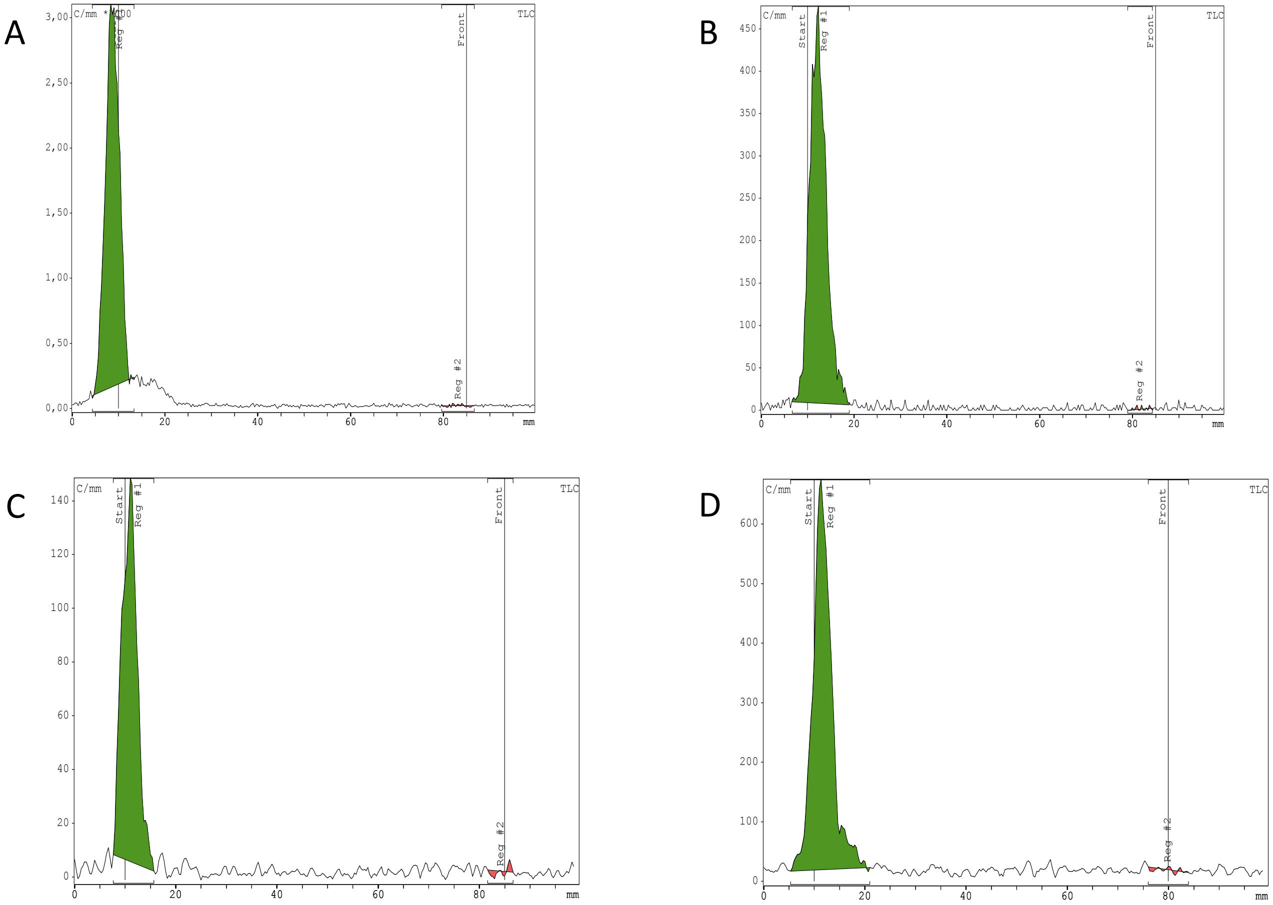

Thin-layer chromatography of ^{99m}Tc-labeled 1-thio-β-D-glucose (A+B) and ^{99m}Tc-labeled 5-thio-D-glucose (C+D) after 24h incubation at 37°C in phosphate-buffered saline (PBS) (A+C) or bovine serum (B+D), activity 10MBq.
The radiochemical purity was >99% in all probes.
