# Supplementary material for: In vivo evaluation of tumor uptake and bio-distribution of 99mTc-labeled 1-thio-β-D-glucose and 5-thio-D-glucose in mice model
Source: EJNMMI Radiopharm Chem. 2024 Mar 29;9:26. doi: 10.1186/s41181-024-00253-3 (PMC10980667; doi:10.1186/s41181-024-00253-3)
Supplement: Supplementary file 4 — Additional file 4. Planar imaging 60min p.i. of 99mTc-labeled 1-thio-β-D-glucose (A-C) and 99mTc-labeled 5-thio-D-glucose (D-F) in C57/BL6 non-tumor-bearing mice. No uptake in the thyroid gland visible. [file 41181_2024_253_MOESM4_ESM.pdf]

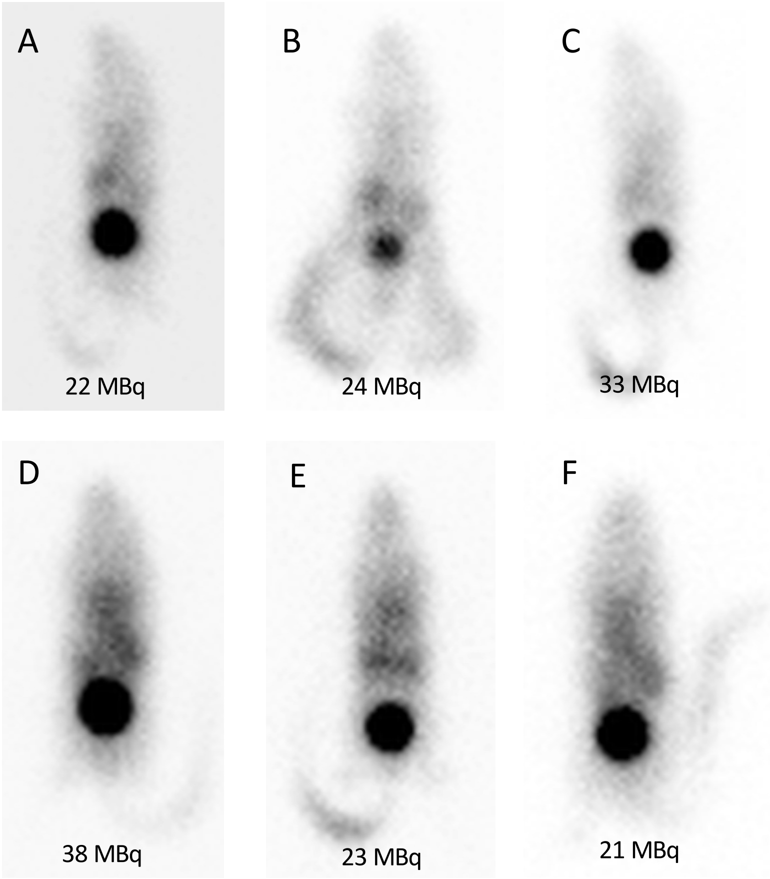

Planar imaging 60min p.i. of ^{99m}Tc-labeled 1-thio-β-D-glucose (A-C) and ^{99m}Tc-labeled 5-thio-D-glucose (D-F) in C57/BL6 non-tumor-bearing mice. No uptake in the thyroid gland visible.
