# Supplementary material for: In vivo evaluation of tumor uptake and bio-distribution of 99mTc-labeled 1-thio-β-D-glucose and 5-thio-D-glucose in mice model
Source: EJNMMI Radiopharm Chem. 2024 Mar 29;9:26. doi: 10.1186/s41181-024-00253-3 (PMC10980667; doi:10.1186/s41181-024-00253-3)
Supplement: Supplementary file 5 — Additional file 5. Organ and tissue uptake by tracer and tumor type. [file 41181_2024_253_MOESM5_ESM.pdf]

Supplemental Table 1a. Organ and tissue uptake of 1-TG in HCT116 model

| **1 TG HCT116** | | | | | | | |
|---|---|---|---|---|---|---|---|
| | 0.5h | | 2h | | 4h | | |
| Organ | ID% | SD | ID% | SD | ID% | SD | p-value |
| Blood | 2.3188 | 1.6806 | 0.9626 | 0.3330 | 0.4022 | 0.1137 | **0.021** |
| Brain | 0.0708 | 0.0543 | 0.0287 | 0.0062 | 0.0264 | 0.0176 | **0.049** |
| Heart | 0.7801 | 0.3014 | 0.4199 | 0.0729 | 0.2473 | 0.0701 | **0.012** |
| Liver | 2.4642 | 2.8502 | 0.9035 | 0.4040 | 0.8242 | 0.5853 | 0.472 |
| Kidney left | 8.9246 | 2.7021 | 10.4170 | 4.5865 | 12.2881 | 5.1901 | 0.500 |
| Kidney right | 8.4938 | 2.7125 | 10.6467 | 4.6504 | 13.3942 | 5.4821 | 0.334 |
| Spleen | 0.8677 | 0.7649 | 0.3871 | 0.1180 | 0.5676 | 0.6594 | 0.245 |
| Stomach | 0.2731 | 0.0613 | 0.2332 | 0.1037 | 0.2376 | 0.1642 | 0.874 |
| Bowel | 0.6638 | 0.3817 | 0.7870 | 0.3463 | 0.4221 | 0.3865 | 0.367 |
| Lungs | 1.2743 | 0.5762 | 0.6840 | 0.1438 | 0.4349 | 0.0446 | **0.021** |
| Muscle | 0.5714 | 0.2224 | 0.1521 | 0.0440 | 0.2003 | 0.1577 | **0.038** |
| Tumor | 0.8564 | 0.2749 | 0.6104 | 0.1648 | 0.5989 | 0.1566 | 0.231 |
| Tumor/Muscle-Ratio | 1.8664 | 1.2971 | 4.1497 | 1.1486 | 4.2251 | 2.7028 | 0.167 |

n=4

Supplemental Table 1b. Organ and tissue uptake of 1-TG in A549 model

**1 TG A549**

| | 0.5h | | 2h | | 4h | | |
|---|---|---|---|---|---|---|---|
| Organ | ID% | SD | ID% | SD | ID% | SD | p-value |
| Blood | 2.3650 | 0.4781 | 1.0228 | 0.4188 | 0.6260 | 0.1231 | **0.015** |
| Brain | 0.0615 | 0.0178 | 0.0427 | 0.0231 | 0.0227 | 0.0082 | *0.062* |
| Heart | 0.9509 | 0.2324 | 0.6232 | 0.2071 | 0.3685 | 0.1173 | **0.023** |
| Liver | 2.7391 | 3.1786 | 3.7832 | 3.3172 | 1.5846 | 0.7279 | 0.874 |
| Kidney left | 11.2703 | 3.2252 | 6.1462 | 3.7154 | 12.0807 | 12.3509 | 0.389 |
| Kidney right | 11.1532 | 3.2931 | 6.2191 | 3.7755 | 11.9807 | 12.4438 | 0.389 |
| Spleen | 0.9946 | 0.7521 | 1.3146 | 1.0719 | 0.5610 | 0.2726 | 0.778 |
| Stomach | 0.3059 | 0.0830 | 0.1354 | 0.0762 | 0.1661 | 0.0949 | *0.069* |
| Bowel | 0.6750 | 0.1422 | 0.5653 | 0.5435 | 0.2330 | 0.1022 | *0.062* |
| Lungs | 1.5995 | 0.5019 | 0.9548 | 0.2763 | 0.6523 | 0.1171 | **0.038** |
| Muscle | 0.3518 | 0.0319 | 0.1487 | 0.0234 | 0.1236 | 0.0428 | **0.020** |
| Tumor | 0.7811 | 0.3587 | 0.4674 | 0.1972 | 0.5185 | 0.3519 | 0.397 |
| Tumor/Muscle-Ratio | 2.2517 | 1.1179 | 3.1543 | 1.1194 | 4.0138 | 1.7816 | 0.210 |

n=4

Supplemental Table 1c. Organ and tissue uptake of 5-TG in HCT116 model

| **5 TG HCT116** | | | | | | | |
|---|---|---|---|---|---|---|---|
| | 0.5h | | 2h | | 4h | | |
| Organ | ID% | SD | ID% | SD | ID% | SD | p-value |
| Blood | 1.7878 | 1.0652 | 0.8067 | 0.1412 | 0.6062 | 0.2559 | **0.015** |
| Brain | 0.0495 | 0.0158 | 0.0366 | 0.0114 | 0.0387 | 0.0309 | 0.667 |
| Heart | 0.9508 | 0.3219 | 0.4194 | 0.0753 | 0.2566 | 0.1230 | **0.012** |
| Liver | 5.8800 | 1.4665 | 8.7182 | 6.4021 | 4.8216 | 1.3143 | 0.367 |
| Kidney left | 6.1779 | 1.0362 | 4.2988 | 1.1155 | 2.8141 | 0.7138 | **0.017** |
| Kidney right | 6.2368 | 0.9311 | 4.3776 | 1.1495 | 2.9333 | 0.4559 | **0.013** |
| Spleen | 2.8531 | 1.8973 | 5.0019 | 3.9424 | 2.9412 | 1.5077 | 0.297 |
| Stomach | 0.7464 | 0.5135 | 0.2231 | 0.0981 | 0.1725 | 0.0837 | 0.105 |
| Bowel | 0.7752 | 0.2843 | 0.7091 | 0.6137 | 0.3147 | 0.2223 | *0.097* |
| Lungs | 10.3817 | 2.0293 | 4.3414 | 4.1510 | 1.9437 | 1.4005 | **0.043** |
| Muscle | 0.7823 | 1.2145 | 0.1381 | 0.0526 | 0.1666 | 0.1655 | 0.309 |
| Tumor | 0.5339 | 0.2509 | 0.3104 | 0.0226 | 0.2709 | 0.0826 | *0.086* |
| Tumor/Muscle-Ratio | 2.1659 | 1.7001 | 2.4634 | 0.7839 | 2.7414 | 1.7891 | 0.981 |

n=4

Supplemental Table 1d. Organ and tissue uptake of 5-TG in A549 model

| **5 TG A549** | | | | | | | |
|---|---|---|---|---|---|---|---|
| | 0.5h | | 2h | | 4h | | |
| Organ | ID% | SD | ID% | SD | ID% | SD | p-value |
| Blood | 1.0807 | 1.1982 | 0.6028 | 0.3704 | 0.4142 | 0.1829 | 0.693 |
| Brain | 0.1609 | 0.1323 | 0.0935 | 0.0525 | 0.0557 | 0.0233 | 0.367 |
| Heart | 0.7435 | 0.5510 | 0.4192 | 0.1295 | 0.3146 | 0.1651 | 0.583 |
| Liver | 6.8791 | 4.1662 | 6.0255 | 2.9446 | 7.0713 | 3.3201 | 0.981 |
| Kidney left | 3.2512 | 1.5003 | 2.3772 | 0.5401 | 1.6884 | 0.7506 | 0.500 |
| Kidney right | 3.3925 | 1.4887 | 2.4264 | 0.5081 | 1.9270 | 1.0136 | 0.500 |
| Spleen | 2.9482 | 3.0580 | 2.6271 | 2.0466 | 4.0604 | 2.5387 | 0.595 |
| Stomach | 0.1802 | 0.1797 | 0.0910 | 0.0112 | 0.4650 | 0.4996 | 0.116 |
| Bowel | 0.5048 | 0.5487 | 0.2542 | 0.1260 | 0.2264 | 0.1373 | 0.874 |
| Lungs | 4.1008 | 2.9607 | 2.4486 | 1.2061 | 2.2072 | 1.7874 | 0.667 |
| Muscle | 0.1449 | 0.1047 | 0.1222 | 0.0525 | 0.0551 | 0.0309 | 0.231 |
| Tumor | 0.2072 | 0.1804 | 0.2211 | 0.0653 | 0.1957 | 0.0973 | 0.550 |
| Tumor/Muscle-Ratio | 1.6754 | 0.8576 | 2.0266 | 0.7108 | 4.0718 | 1.2647 | **0.023** |

n=4
