# Supplementary material for: In vivo evaluation of tumor uptake and bio-distribution of 99mTc-labeled 1-thio-β-D-glucose and 5-thio-D-glucose in mice model
Source: EJNMMI Radiopharm Chem. 2024 Mar 29;9:26. doi: 10.1186/s41181-024-00253-3 (PMC10980667; doi:10.1186/s41181-024-00253-3)
Supplement: Supplementary file 6 — Additional file 6. Radiolabeling process. [file 41181_2024_253_MOESM6_ESM.docx]

Supplementary data – Radiolabeling:

For labeling stannous chloride has been added as reducing agent (0.35 μmol in 0.1 N HCl, Sigma-Aldrich®) within 10 minutes at 20° C under nitrogen atmosphere. 250 μl phosphate-buffered saline (PBS) hase been used to adjust the pH value to 7.4. High-pressure liquid chromatography (HPLC, Agilent Technologies®, HPLC 1200er series, Santa Clara, USA) and thin-layer chromatography (TLC, Raytest®, Mini GITA Star, Straubenhardt, Germany) has been utilized to confirm the radiochemical purity and in vitro stability by using aluminium-backed TLC plates (Merck©, Whitehouse Station, USA) and methyl ethyl ketone (MEK, Merck®) as a solvent. HPLC was performed by gradient separation (0-10 min A:B = 100:0; 10-20 min A:B = 50:50; 20-30 min A:B = 0:100; A: water, 0.1% tri- fluorine acetic acid; B: acetonitrile, 0.1% trifluoroacetic acid) on a C18 column (Phenomenex®, Kinetex 2.6 μm C18, 100 x 4.6 mm, Torrance, USA) at a flow rate of 1 ml/min. Then, UV absorption (λ=254 nm) and the synchronized radioactivity detector (Raytest®, GABI Star) has been used for the analysis.
